# Death from Starvation

**Published:** 1920-10-30

**Authors:** Ronald Campbell Macfie


### DEATH FROM STARVATION.

By RONALD CAMPBELL MACFIE, M.A., M.B., C.M., LL.D.

The Lord Mayor of Cork and the other Irish hunger-strikers caused much surprise by the length of time they survived on a diet of nothing but water, and many have gone so far as to affirm that they have been surreptitiously fed. It has been reported, indeed, in the papers that various medical experts have declared it impossible for any man to live without food so long as the Irish hunger-strikers have already lived.

But, physiologically and scientifically speaking, there is nothing very surprising about the long fast of these men, and the surprising thing is that any medical or scientific man should declare it impossible. It is true that, strictly speaking, deaths from starvation have seldom or never occurred, that scientific experiments in fasting have been rarely carried out, and that our clinical facts are comparatively few, but such clinical facts as we do possess certainly point to the possibility of very long fasts, and all physiological facts relevant to the question point in the same direction .

### The Clinical Data.

Let us look first at some of the clinical data at our disposal. We have the cases of experimental fasting by human beings. Succi fasted thirty days on several occasions, once forty days, and once forty-five days; Jacques on different occasions fasted thirty, forty-two, and fifty days; Dr. Tanner fasted forty days; Merlatti fasted fifty days; Gayer fasted forty days; Sevanzin fasted thirty-one days. We have the case of the French convict, certified by Bérard, who lived for sixty-three days without food. We have also the experiments made on dogs by Luciani, Aurowow, Kumagawa, Falck, Howe Mattill, and Hawk, where dogs lived sixty, ninety-eight, a hundred and four, and a hundred and seventeen days without food.

### Medical Supervision.

Most of these fasts have been performed under strict medical supervision, and they certainly go to show that animals can live for long periods without food, and that starvation is a very slow process. Even after fasts of thirty, forty, or fifty days the professional fasters had reserves of food in their tissues, had a fair amount of physical and mental energy, and were very far from moribund. Levanzin, who carried out an experimental fast at the Carnegie Institute in 1912, showed little mental or physical deterioration at the end of the fast, and was very unwilling to terminate it. Even on the final day of the fast he was able to walk upstairs without holding to the banisters. Up to the last day of his fast he went for drives and enjoyed them, and on the last day of his fast he had an audience with a number of medical men, "talked very rapidly and in a lively manner for nearly forty minutes, setting forth his views in a more or less lucid manner." He was not markedly emaciated, though he had lost about 30 lb. in weight.

The Scotch collie "Oscar," which holds the record with the fast of 117 days, also retained energy in a remarkable manner; he was able to jump out of his cage up to the 101st day of his fast; and up to the end of the experiment he continued to bark and to wag his tail vigorously. After the experiment he was sent to a farm, and seemed to be stronger, more energetic, and in better all-round physical condition than before the fast, and subsequently performed another fast of 104 days. The experimenters considered he could live 130 days without food.

Physiologically speaking, as we have already said, there is nothing surprising about these long fasts; for during a fast the vital organs turn cannibal and feed on tissues such as fat and muscular protein which are not essential to life. If the body be kept at rest and warm there will be little food required for purposes of heating and voluntary motion, and the food supplies of the body will be almost entirely at the disposal of the vital organs. The most essential vital organs are the heart and brain, and so long as these continue their reciprocal functions and there is interchange of gases in the lungs, so long will an animal be alive. But the brain and heart require very little food even in normal conditions; and, since during a fast their activity is greatly reduced, they require during a fast even less food still.

### The Amount of Wasting.

We have clinical facts to show just how much food is used by a fasting animal. Thus we find that Succi, starting a forty days' fast at a weight of 55.80 kilograms, finished the fast at a weight of 41.70 kilograms, showing a total loss of 14.10 kilograms—about 25 per cent. of his initial body weight. In a fast of forty-five days he lost 19.34 kilograms. In three fasts of thirty days he lost, on an average, 13.3 kilograms—21.6 per cent. of his initial body weight. In thirty days Levanzin lost 12.95 kilograms—21.4 per cent. of his body weight. In thirty days Jacques lost 10.32 kilograms—16.6 per cent. of his body weight. In the same space of time Gayer lost 17.4 per cent. of his body weight, and Penny 19 per cent. Some of these figures may not be strictly reliable, but they are sufficient to show that a fasting man can live for thirty days at a cost of about 20 per cent. of his body weight.

### Economy in Energy.

The dog "Oscar" lost 62.9 per cent. of its weight in its 117 days' fast; while a dog, which received neither food nor water, lost in sixty-six days about the same percentage of weight and then died. All these facts go to suggest that man has in his own tissues sufficient food material to feed his vital organs for two or three months under the conditions of the experiments. Exactly how long a faster lives will depend partly on the amount of his food reserves, and partly on the rate at which he consumes them; and it must be noticed that in none of the experiments hitherto carried out have expenditure of energy and consequent consumption of tissue food been cut down to irreducible minima. The professional fasters sat upright, climbed stairs, carried on animated discussions, dressed, undressed, bathed, gripped dynamometers, wrote long letters, went for drives, and were exposed to a temperature far below 98° F. In all these directions there was room for considerable economy. It is well known that a man sitting or standing burns away faster than lying, and that a little walking exercise greatly increases his metabolism. Even writing increases tissue waste. Accurate observations were made in the case of Levanzin, and the report states : "The work of writing, therefore, produced a distinct increase of metabolism, which is shown not only by the increase in the carbon-dioxide production and the oxygen consumption, but also by an increase in the respiration rate, the ventilation

41135A

**Death from Starvation**—(*continued*).

of the lungs and the volume per inspiration. Finally, there was a regular and distinct increase in the pulse-rate."

There can be no doubt that the consumption of the fasters' tissue-protein might have been considerably less if the fasters had been kept continually in a lying position.

But still more economy might have been effected by keeping the fasters in a moist atmosphere heated to 97° or 98° F. It is essential to life that the body should be maintained at a temperature of about 97° or 98° F., and if the external temperature be low, the body has to burn more of its own fuel to maintain its temperature; and residence in a room kept at a temperature of 97° F. would markedly reduce tissue waste. It is notable that hibernating animals who are undergoing prolonged fasts at once reduce their heat production, so that their temperature falls to the temperature of the surrounding medium; and it is notable, too, how, if heat production is reduced by a warm environment, appetite and energy fail.

### Warm and Damp Air.

A faster kept in bed in a warm, damp atmosphere would probably easily beat all former fasting records. How long under such conditions a faster might live it would be difficult to say; but it is quite possible that vitality might be reduced to such a low ebb that the daily loss of weight might be very small.

Former fasters, fasting under comparatively unfavourable conditions, have lived forty-five days at the expense of about 25 per cent. of their initial body-weight, and theoretically speaking—even without making allowance for diminution in food consumption with increasing weakness—they have had still enough food in their body to carry them at least another forty-five days, and there can be little doubt that under more favourable circumstances the food on a man's bones might be made to feed his heart and brain for a week or two longer—say for a hundred days.

It all depends upon what reduction of vitality is compatible with its continuance, and there is some evidence to show that life can be almost entirely suspended without death ensuing. There have been cases of cataleptic trance where circulation and respiration have been imperceptible, and where the trance has been mistaken for death. So little oxygen does a hibernating bat require, that it can be kept for a quarter of an hour under water without danger to its life; and a hibernating marmot can live for four hours in an atmosphere of carbon dioxide. There can be little doubt, too, that some Fakirs have the power of suspending their vital functions so far that they can continue to live in closed coffins for some weeks without perceptible heart-beat or respiration.

### The Real Cause of Death.

If life can be reduced to a low enough ebb it may be maintained for very long periods, and there is really nothing very extraordinary in the prolongation of the lives of the Irish hunger-fasters who carried out their fasts under tragical and yet physiologically speaking favourable conditions. It is interesting, however, to remember that the Irish have more than once been almost exterminated by famine, and therefore the survivors have been selected by famine and may have special power of resisting death from starvation.

It does not, however, necessarily follow that because a man dies during a fast he has died of starvation. Very few, if any, men have actually died of starvation. A man who dies in the course of a fast is more likely to die of exposure to cold, or of exertion beyond his limited strength, or of some disorder such as scurvy due to lack of some vitamine.

